# HPLC-PDA/ESI-MS Analysis of Phenolic Compounds and Bioactivities of the Ethanolic Extract from Flowers of Moroccan *Anacyclus clavatus*

**DOI:** 10.3390/plants11243423

**Published:** 2022-12-08

**Authors:** Mounia Chroho, Mustapha Aazza, Aziz Bouymajane, Yassine Oulad El Majdoub, Francesco Cacciola, Luigi Mondello, Touriya Zair, Latifa Bouissane

**Affiliations:** 1 Molecular Chemistry, Materials and Catalysis Laboratory, Faculty of Sciences and Technologies, Sultan Moulay Slimane University, BP 523, Beni-Mellal 23000, Morocco; 2 Research Team Chemistry of Bioactive Molecules and Environment, Laboratory of Innovative Materials and Biotechnologies of Natural Resources, Faculty of Sciences, Moulay Ismail University, BP 11201, Meknes 50070, Morocco; 3 Laboratory of Chemistry-Biology Applied to the Environment, Faculty of Sciences, Moulay Ismail University, BP 11201, Meknes 50070, Morocco; 4 Team of Microbiology and Health, Laboratory of Chemistry-Biology Applied to the Environment, Faculty of Sciences, Moulay Ismail University, BP 11201, Meknes 50070, Morocco; 5 Department of Chemical, Biological, Pharmaceutical and Environmental Sciences, University of Messina, 98168 Messina, Italy; 6 Department of Biomedical, Dental, Morphological and Functional Imaging Sciences, University of Messina, 98125 Messina, Italy; 7 Chromaleont s.r.l., c/o, Department of Chemical, Biological, Pharmaceutical and Environmental Sciences, University of Messina, 98168 Messina, Italy; 8 Department of Sciences and Technologies for Human and Environment, University Campus Bio-Medico of Rome, 00128 Rome, Italy

**Keywords:** *Anacyclus clavatus*, flower ethanolic extract, total polyphenol content, flavonoid content, phenolic composition, FRAP activity, antibacterial activity

## Abstract

In this work, polyphenols were extracted from *Anacylus clavatus* flowers using a hydroethanolic solvent, and the obtained extract was studied for its total phenol and flavonoid contents and evaluated for its antioxidant and antibacterial capacities. The contents of total phenols and flavonoids were measured by employing gallic acid and quercetin as references, respectively, and the phenolic composition analysis was conducted using high-performance liquid chromatography combined with a photodiode array and electrospray ionization mass spectrometry (HPLC-PDA/ESI-MS). The antioxidant capacity of the extracts was tested using a potassium ferric reducing antioxidant power (PFRAP) assay, and the antibacterial activity assay was carried out against Gram-negative bacteria (*Escherichia coli* and *Salmonella typhimirium*) and Gram-positive bacteria (*Staphyloccocus aureus* and *Listeria monocytogenes*) using the broth microdilution assay. The phenolic and flavonoid contents of the extracts equaled 9.53 ± 0.48 mg GAE/g dm and 1.31 ± 0.06 mg QE/g dm, respectively. The chromatographic analysis of the phenolic profile detected 26 phenolic compounds belonging to phenolic acids, flavones and flavonols, and with the caffeoylquinic acid derivatives being the major phenolic compounds present in 12 isomers. Only one organic compound, *viz.* citric acid, was found. The extracts exhibited interesting antioxidant activity. Bacteriostatic activity towards *Escherichia coli* and bactericidal activity against *Salmonella typhimirium*, *Staphyloccocus aureus* and *Listeria monocytogenes* were determined. This study revealed that *Anacyclus clavatus* flower extracts contain phenolic compounds with interesting bioactivities.

## 1. Introduction

One of the largest families of flowering plants in the world is the Asteraceae family, popularly known as the sunflower family. It comprises more than 1600 genera and 25,000 species with incredibly varied morphologies, ranging from 1-cm-tall herbs to almost 30-m-tall trees [1,2]. Asteraceae plants have been distinguished by their capitula, which include closely arranged flowers on a receptacle encircled by bracts [3]. Asteraceae family plants are distributed all over the world, except for Antarctica, and are most prevalent in subtropical, arid and semi-arid climates [1,4]. Most plants of the Asteraceae family have been used traditionally in conventional medicine; the medicinal use of some of them has been known for more than 3000 years [5]. The pharmacological properties of Asteraceae species have been related to the presence of several phytochemical components, including polyphenols, flavonoids, essential oils, sesquiterpene, lignans, lactones and saponins [1,5].

*Anacyclus* from the Asteraceae family comprises about 12 species distributed in the Mediterranean countries (North Africa, Southern Europe and the Middle East) [6,7]. Some of them are known as chamomile plants (*Anacyclus pyrethrum* and *Anacyclus clavatus*) [6,8,9]. *Anacyclus* species are annual herbs with a short vegetative cycle and a blooming period between April and June; they are employed in traditional medicine due to their demonstrated biological activities. Extracts from the aerial parts of *Anacyclus maroccanus* and *Anacyclus radiatus* were found to have interesting hypoglycemic, antiglycative, radical scavenging and antibacterial activities [6]. The *Anacyclus valentinus* essential oil has showed antifungal activity [10]. Finally, the most researched species of *Anacyclus*, *Anacyclus pyrethrum*, has showed several interesting biological activities, including anticonvulsant, anxiolytic [11], anabolic, aphrodisiac [12] and immunostimulating [13] properties.

*Anacyclus clavatus*, also known as *white anacyclus* [14] or chamomile, is an annual, spontaneous and abundantly present herb in the Mediterranean countries. It is 20 to 50 cm high with a flower head with two different kinds of flowers: yellow flowers tubulated in the interior and white flowers ligated around the yellow ones. The flowers are grouped in 2.5–3-cm-long rayed terminal capitula. Flowering starts at the end of March and extends to May, and is particularly advantageous during the rainy season [15,16]. The aerial parts of this plant are used as herbal teas to traditionally treat some ailments, especially those related to the gastric system (gastric ulcers, stomach pain, digestive disorders and bloating). Its essential oil and extracts present important activities, including antioxidant [7,17,18], anti-inflammatory [7] and antimicrobial activities [16]. *Anacyclus clavatus* extracts have been reported to be rich in phenolic compounds [7]. The yield of *Anacyclus clavatus* essential oil via extraction is very low. Boudihar et al. cited an essential oil yield range of 0.015% to 0.019% depending on the part of the plant and the method of extraction used. The flowers’ essential oil consists mainly of β-thuyone, while the composition of the essential oil from the leaves and stems consists of germecrene as the major constituent in addition to δ-elemene and α-caryophyllene [17,19]. Another study conducted by Hammami et al. found that the major components of the *Anacyclus clavatus* essential oil were trans-chrysanthenyl acetate, cis-thujone, chrysanthenone and trans-thujone [16].

*Anacyclus clavatus* has been cited in several studies conducted on the flora of Morocco, in regions of Marrakech [20], in the Moroccan Middle Atlas region (Sefrou, Mazdou, Timahdite [21]) and in the central plateau of Morocco (Aguelmous, Moulay Bouazza, Tiddas [22]). The *Anacyclus clavatus* species is not well documented, and to the best of our knowledge no previous studies have investigated the composition and biological activities of *Anacyclus clavatus* from Morocco.

This study aims to estimate the phenolic content and evaluate the phenolic profile of the hydroethanolic extract from flowers of *Anacyclus clavatus* harvested in the Khenifra region (Middle Atlas region of Morocco) and to evaluate its antioxidant and antibacterial activities.

## 2. Results and Discussion

### 2.1. Extraction Yield of Polyphenols

The crude hydroethanolic extract of *Anacyclus clavatus* flowers was obtained with an extraction yield of 35.03% (Table 1). This value was much higher than those reported by Bouriche et al. for methanolic and aqueous extracts of *Anacyclus clavatus*. The extracts were prepared via maceration and the yields reported were respectively 19% and 17% [7].

For other species of *Anacyclus*, the *Anacyclus valentinus* methanol extract presented an extraction yield of 17.82 ± 0.49 [23]. The *Anacyclus pyrethrum* root ethanolic extract gave a 3.56% (*w*/*w*) yield [11]. The ethanolic extracts from different parts of two species of *Anacyclus pyrethrum* presented yields ranging from 6% to 16%, whereby the highest yields were related to the root extracts [24]. Several studies have concluded that the solvent and extraction technique that are employed affect the extraction yield, in addition to the differences in phenolic composition between species and parts of the plant [25].

### 2.2. Contents of Total Phenols (TPC) and Flavonoids (FC)

The total phenolic and flavonoid contents of the ethanolic extract from *Anacyclus clavatus* flowers were respectively 9.53 ± 0.48 mg GAE/g dm (milligrams of gallic acid equivalent per gram of dry matter) and 1.31 ± 0.06 mg QE/g dm (milligrams of quercetin equivalent per gram of dry matter) (Table 1). For the same plant, Bouriche et al. obtained the values for the methanolic extract (TPC = 131.30 ± 6.88 mg GAE g^–1^ extract; FC = 9.96 ± 0.43 mg QE g^–1^ extract) and aqueous extract (TPC = 79.06 ± 3.24 mg GAE g^–1^ extract; FC = 16.39 ± 1.38 mg QE g^–1^ extract) [7].

The methanolic extract of *Anacyclus valentinus* presented a TPC and FC of 115.47 ± 0.13 mg GAE/g and 52.15 ± 0.78 mg QE/g, respectively [23]. Sissi et al. studied methanol and ethyl acetate extracts of *Anacyclus radiatus* and *Anacyclus maroccanus,* highlighting how significant amounts of flavonoids and polyphenols, particularly those made from *Anacyclus radiatus*, occur in the extracts. Additionally, methanolic extracts have been reported to present higher amounts than ethyl acetate extracts [6]. Erez et al. studied the phenolic and flavonoid contents for extracts from the leaves and flowers of *Anacyclus anatolicus*, and for every solvent (acetone, ethanol and water) the extract from the flowers presented the highest phenolic and flavonoid contents [26].

### 2.3. Identification of Phenolic Profile via an HPLC-PDA/ESI-MS Analysis

**Figure 1:**
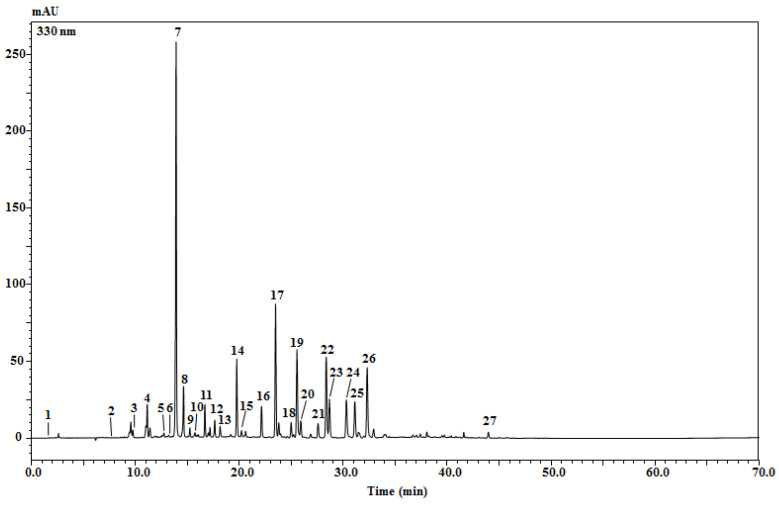
Chromatographic profile of phenolic compounds in *Anacyclus clavatus* flower extract (EtOH:H_2_O 7:3 *v*/*v*) recorded at 330 nm.

The use of high-performance liquid chromatography combined with a photodiode array and electrospray ionization mass spectrometry (HPLC-PDA/ESI-MS) revealed 26 phenolic compounds in the crude ethanolic extract of *Anacyclus clavatus* flowers (Figure 1). Sixteen of them were phenolic acids, while the rest were flavonoids (flavones and flavonols), while only one organic acid, citric acid, was found. The caffeoylquinic acid derivatives are the major phenolic compounds in the extract (Table 2). They are present in 11 isomers for both classes of mono-caffeoylquinic and di-caffeoylquinic acids. The flavonoids are present as flavones (myricetin-hexoside, luteolin-dihexoside, apigenin-7-O-diglucuronide, apigenin-7-glucuronosyl-glucoside, apigenin-7-O-glucoside, apigenin 7-O-glucuronide, apigenin and quercetin O-hexoside) and flavonols (kaempferol-7-O-glucoside and patuletin 3-O-glucoside).

It is to be noted that chemical composition of *Anacyclus clavatus* differs from those reported by Bouriche et al. [7] for methanol and aqueous extracts of *Anacyclus clavatus* from Algeria. The authors reported the presence of gentisic acid, chlorogenic acid, 4-hydroxybenzoic acid, protocatechuic acid, caffeic acid, syringic acid, vanillic acid, rutin, quercetin-3-β-D-glucoside, sinapic acid, naringin, polydatine, diosmin, apigetrin, cinnamic acid and apigenin.

Caffeoylquinic acid derivatives are a significant family of secondary metabolites and phenolic acids. They can be found in large quantities in a wide range of plants, as well as in a variety of foods, including vegetables, fruits, coffee and spices. In the plant kingdom, they have been found in a variety of plant families, including the Asteraceae family. Caffeoylquinic acids consist of a quinic acid group and one to four caffeoyl group residues, respectively named mono-, di-, tri- or tetra-caffeoylquinic acid [33,34,35].

The caffeoylquinic acid derivatives present in the studied extract are phenolic acids, as previously reported in the phenolic profile of *Anacyclus* species. 3-O-caffeoylquinic acid was reported as the major compound in the phenolic composition of *Anacyclus anatolicus* extracts from leaves and flowers [26]. Di-acyl caffeoylquinic acid was also identified in *Anacyclus pyrethrum* roots [36], along with different caffeoylquinic acid derivates occurring in the aqueous and methanol extracts of *Anacyclus pyrethrum* [37].

The other phenolic acids, coumaroylquinic acid and feruloylquinic acid, were identified in the phenolic compositions of some Asteraceae plants [5].

Concerning the flavonoid contents, *Anacyclus* species have been demonstrated to be rich in flavonoids. The methanol extract (at 80%) of *Anacyclus valentinus* has been reported to contain myricetin, quercetin, kaempferol, luteolin and apigenin [23]. These flavonoids, in addition to galangin, rutin, acacetin, hesperidin, pinocembrin and chrysin, were identified in the 95% (*v*/*v*) ethanol extract of the same plant, *Anacyclus valentinus* [38]. Patuletin has been isolated from the ray flowers and yellow discs of *Anacyclus radiatus* [39].

### 2.4. Antioxidant Capacity of Anacyclus clavatus Flower Extract Tested Using the Potassium Ferric Reducing Antioxidant Power (PFRAP) Assay

**Figure 2:**
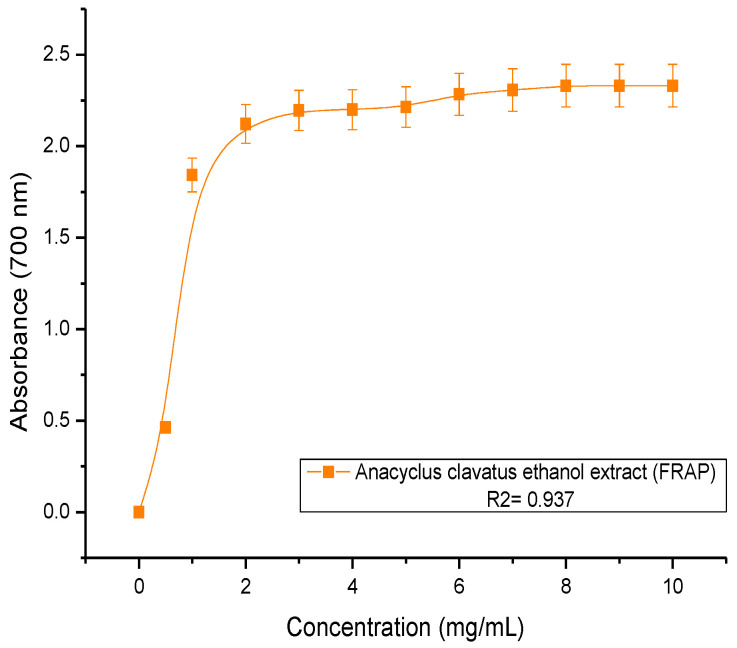
Antioxidant capacity of the ethanolic extract of *Anacyclus clavatus* flowers assessed using a PFRAP assay.

The ethanolic extract of *Anacyclus clavatus* flowers displayed important antioxidant activity (Figure 2). The EC_50_ parameter was found to equal 0.91 ± 0.04 mg/mL. The EC_50_ parameter of the ascorbic acid tested under the same condition was found to equal 0.031 mg/mL (Table 1).

The antioxidant capacity of *Anacyclus clavatus* extract has been previously tested. Boudihar et al. investigated several extracts, using methanol, ethanol and acetone as solvents, from different parts of the plant (leaves and stems and flowers). Their results proved that all extracts exhibited antioxidant power. For the extracts from the flowers, the methanolic one was the most effective, while for the extracts from the leaves and stems, the acetone extract turned out to be the most powerful one [17]. The antioxidant power of aqueous extract from *Anacyclus clavatus* has also been proven [7].

The antioxidant capacity of the other species of *Anacyclus* was also investigated. Acetone, ethanol and aqueous extracts from *Anacyclus anatolicus* leaves and flowers [26]; methanolic and ethyl acetate extracts from *Anacyclus radiatus* and *Anacyclus maroccanus* [6]; and aqueous and methanol extracts of *Anacyclus pyrethrum* [37] all exhibited antioxidant effects.

Some literature reports have identified caffeoylquinic acid derivatives as the primary antioxidant metabolites frequently identified from plants belonging to Asteraceae species [26,34]. The traditional therapeutic use of plants containing caffeoylquinic acid derivatives may be highly related to the antioxidant power of these metabolites. Being electron-rich compounds, they may be oxidized in vivo into quinoids and may potentially present disease-modifying properties [33]. The correlations among phenolic compositions and the samples’ antioxidant activity levels were verified in several plants extracts and products [40,41,42].

### 2.5. Antibacterial Capacity of Anacyclus clavatus Flower Extract

Investigations on the antibacterial power of *Anacyclus clavatus* flower ethanol extract have been carried out towards Gram-negative bacteria (*Escherichia coli* and *Salmonella typhimirium*), and Gram-positive bacteria (*Staphyloccocus aureus* and *Listeria monocytogenes*) using a broth microdilution assay. The tested concentrations for the MIC and MBC were 0.32, 0.65, 1.30, 2.60, 5.20, 10.41, 20.83, 41.66, 83.33 and 166.66 mg/mL. The MIC values of the *Anacyclus clavatus* flower extract towards all tested bacteria varied between 20.83 ± 0.12 and 41.66 ± 0.15 mg/mL. The extract exhibited bacteriostatic properties towards *Escherichia coli* (MBC/MIC = 8) and bactericidal activity against *Salmonella typhimirium*, *Staphyloccocus aureus* and *Listeria monocytogenes* (MBC/MIC = 4 and 2) (Table 3).

*Anacyclus pyrethrum* is by far the most studied *Anacyclus* species for its antibacterial activity. Jawhari et al. studied ethanolic extracts from different parts of two varieties of *Anacyclus pyrethrum*. The authors concluded that all studied extracts were discovered to have a mostly bactericidal effect, and that differences exist between strains, as well as according to the extract and the part of the plant used. In accordance with our results, a ethanolic extract of *Anacyclys pyrethrum* var *depressus* from capitula was found to be bactericidal towards *Staphyloccocus aureus,* while *Escherichia coli* was resistant to it [24]. The results achieved in this work are in agreement with the antimicrobial properties mentioned by Sissi et al., who found that the methanolic extracts from *Anacyclus radiatus* and *Anacyclus maroccanus* presented lower potency towards *Escherichia coli* [6]. Selles et al. also reported similar results for a methanolic extract of *Anacyclus pyrethrum* from aerial parts, which was able to inhibit *Staphyloccocus aureus*, despite slightly affecting *Escherichia coli* [43].

## 3. Materials and Methods

### 3.1. Plant Material

**Figure 3:**
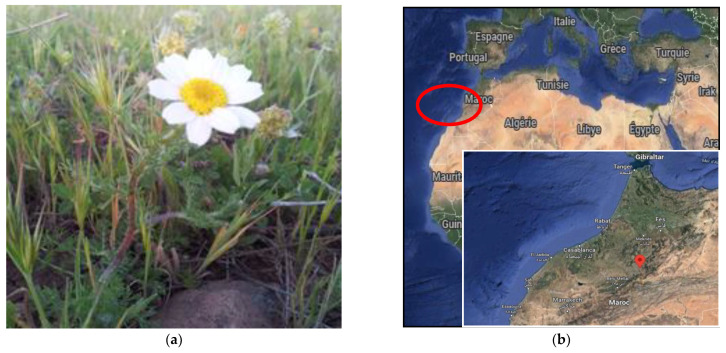
(**a**) Picture of *Anacyclus clavatus* and (**b**) localization of the site of harvest.

The flowers of *Anacyclus clavatus* were harvested in the Middle Atlas are of Morocco in the El Hammam region (Khenifra province) (Figure 3) in late May. The plant grows spontaneously in the site and was collected, cleaned and dried by the El Hammam Cooperative for the Valorization of Medicinal and Aromatic Plants. The site of the harvest is located at heights starting at 1186 m. The plant was identified at the Scientific Institute of Rabat, and was crushed to a fine powder in order to perform the polyphenol extraction.

### 3.2. Extraction of Polyphenols from Anacyclus clavatus Flowers

The Soxhlet method was used for the extraction of polyphenols from *Anacyclus clavatus* flowers. Here, 30 g of dried *Anacyclus clavatus* flowers as a fine powder was extracted using aqueous ethanol (70%). Several cycles were performed until the depletion and discoloration of the plant powder were achieved. Following filtration, the solvent was evaporated under vacuum conditions from the filtrate. The resulting residue was the crude ethanolic extract. The yield of the crude extract was computed as follows:

$$Y\%=\frac{\mathrm{Mass}\;\mathrm{of}\;\mathrm{crude}\;\mathrm{extract}}{\mathrm{Mass}\;\mathrm{of}\;\mathrm{dry}\;\mathrm{matter}\;\mathrm{powder}}\times 100=\frac{m_{0}}{30}\times 100$$

### 3.3. Estimation of Total Phenol and Flavonoid Contents in Hydroethanolic Extract of Anacyclus clavatus Flowers

The estimation of the TPC in the given sample was conducted using the Folin–Ciocalteu method [44]. This consisted of mixing a quantity of the extract with 5 mL of 10% Folin–Ciocalteu reagent, then 1.5 mL of aqueous sodium carbonate Na_2_CO_3_ at 7.5% (*m*/*v*) was added. The solutions were adjusted to 100 mL with distilled water. Then, they were immediately homogenized and maintained at room temperature for 30 min in the dark. The absorbance of the solution was measured at 760 nm.

**Figure 4:**
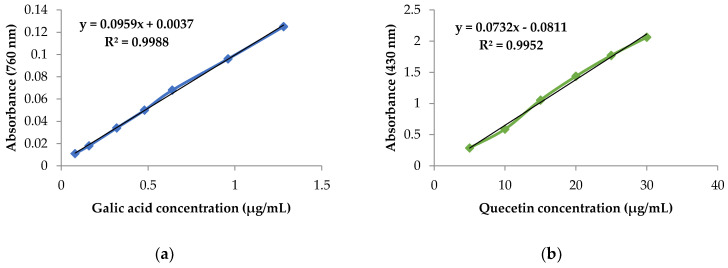
Calibration curves of (**a**) gallic acid and (**b**) quercetin.

By employing the same operating approach, gallic acid was used as a positive control and its calibration curve was assessed (y =0.095x +0.003) (Figure 4a). The results are expressed as milligrams of gallic acid equivalent per gram of dry matter (mg GAE/g dm).

$$T=\frac{C\times V}{m\;\left(\mathrm{dry}\;\mathrm{matter}\right)}\times D$$

where C is the concentration determined by the regression equation of the gallic acid calibration, D is the dilution factor and V is the sample volume.

The FC was estimated using a method involving aluminum trichloride (AlCl_3_) [45]. It consisted of preparing a solution with a volume of the extract and 20 mL of distilled water, which were then mixed with 0.1 mL of AlCl_3_ at 10% (*m*/*v*). The mixture was diluted with methanol (50 mL), quickly homogenized and then stored at room temperature for two hours in the dark. The absorbance was measured at 430 nm.

The same operating approach was employed for quercetin, and its calibration curve was assessed (y =0.073x−0.081) (Figure 4b). The results are expressed as milligrams of quercetin equivalent per gram of dry matter (mg QE/g dm).

### 3.4. Identification of the Phenolic Composition of the Extract via Chromatographic Analysis Using HPLC-PDA-ESI-MS

#### 3.4.1. Sample Preparation

The crude ethanolic extract of *Anacyclus clavatus* flowers was redissolved in ethanol. It was diluted at 1:40 (*v*/*v*). For the chromatographic analysis, an injection volume of 5 µL was used, and the analysis was performed in triplicate.

#### 3.4.2. HPLC-MS Conditions

The material and conditions for the HPLC-MS analysis were the same as described in previous studies [46,47].

#### 3.4.3. Standard Employed

Three polyphenolic standards (rutin, quercetin and kaempferol-3-glucoside) were used to quantify the amounts of polyphenols in the sample extract. Each analysis was performed in 6 replicates of the same preparation. The data acquisition was performed using the software program Shimadzu LabSolution ver. 5.99.

### 3.5. Antioxidant Capacity of the Extract Assessed Using Potassium Ferric Reducing Antioxidant Power (PFRAP) Assay

The PFRAP assay is a single electron transfer (SET) reaction based method for determining antioxidant activity. It is a simple, quick and economic technique that uses potassium ferricyanide K_3_Fe(CN)_6_ as a ferric reagent (Fe^3+^) that can be reduced by antioxidants present in the plant extract to potassium ferrocyanide (Fe^2+^). The end result of the reducing power of the tested antioxidant is Prussian blue, which can be measured spectrophotometrically. The test is conducted in acidic pH conditions to maintain iron solubility [46,48,49].

The PFRAP test was performed according to the method described by Koncic [45]. Different extract concentrations between 0 and 5 mg/mL were prepared. Next, 2.5 mL of a phosphate-buffered solution (0.2 M, pH 6.6) was mixed with 0.5 mL of each concentration and 2.5 mL of 1% solution of K_3_Fe(CN)6. The obtained solutions were kept in a water bath at 50 °C for a period of 20 min. Then, 2.5 mL of trichloracetic acid (10%) was added to stop the reaction. Afterwards, the mixtures were centrifuged for 10 min at 3000*× g*. Finally, 2.5 mL of distilled water in addition to 0.5 mL of an aqueous solution of FeCl_3_ at 0.1% was mixed with 2.5 mL of the supernatant at each concentration. Measurements of the absorbance at 700 nm were then taken. Increased absorbance in the reaction medium denotes an increase in the sample’s reducing power.

**Figure 5:**
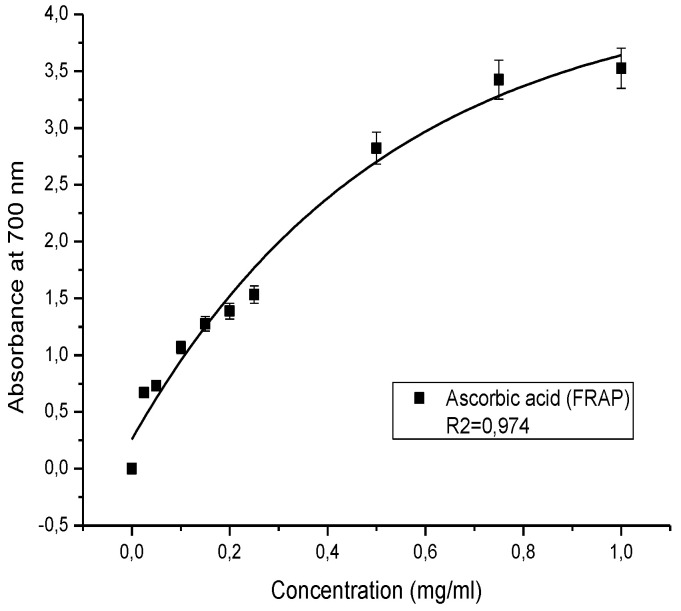
Antioxidant capacity of ascorbic acid as assessed by PFRAP assay.

Ascorbic acid (vitamin C), which is an important physiological antioxidant abundant in many plants, was used as a positive control, and its absorbance was measured under identical circumstances for as the sample (Figure 5).

The antioxidant capacity was expressed by the “effective concentration (EC_50_)” parameter, which corresponds to an absorbance equal to 0.5. This parameter allows a comparison of the reducing activity levels of the control and the sample.

### 3.6. Antibacterial Activity

#### 3.6.1. Bacterial Strains and Their Conditions of Growth

Four bacterial strains (*Salmonella typhimirium*, *Escherichia coli*, *Listeria monocytogene* and *Staphyloccocus aureus*) were used to evaluate the antibacterial activity of the *Anacyclus clavatus* flower ethanolic extract. Bacterial strains obtained from the frozen stock (−80 °C) were distributed on Mueller–Hinton agar, which were kept at 37 °C for one day (24 h). Next, bacterial suspensions were made in sterile distilled water and calibrated to the equivalent of 0.5 McFarland standards (108 cfu/mL).

#### 3.6.2. Broth Microdilution Method

The MIC (minimum inhibitory concentration) and MBC (minimum bactericidal concentration) of the ethanolic extract from *Anacyclus clavatus* flowers were assessed using the broth microdilution method, as described in a previous study [46].

### 3.7. Statistical Analysis

The means ± the standard error of the mean were used to express the gathered results. A one-way analysis of variance (ANOVA) was used in the statistical analysis of the antioxidant capacity using the SPSS and Origin software programs. The differences were deemed significant at p ≤ 0.05 and each experiment was carried out three times.

## 4. Conclusions

In the present study, the hydroethanolic extract from the flowers of *Anacyclus clavatus* from the Middle Atlas region of Morocco was characterized for its phenolic profile and evaluated for its antioxidant and antibacterial properties. The results showed that a total of 26 phenolic compounds were identified in the *Anacyclus clavatus* flower extract. The caffeoylquinic acid derivatives were the major phenolic compounds present in 12 isomers, whose molecular speciation, as assessed by analyzing the respective MS fragmentation patterns, will be elucidated within the framework of our future work. This extract turned out to possess remarkable antioxidant and antibacterial capacities, which may be due to the presence of major compounds in the extract, including apigenin, caffeoylquinic acid and quercetin derivatives. The obtained findings could be useful for further studies on *Anacyclus clavatus* extract for its use in the food and pharmaceutical fields.

## Figures and Tables

**Table 1:** Extraction yield, contents on total phenols (TPC) and flavonoids (FC) and antioxidant capacity of ethanolic extract from *Anacyclus clavatus* flowers. Values are expressed as the means ± SD.

Extraction Yield	Total Phenol Content (TPC)	Flavonoid Content (FC)	EC50 Extract (PFRAP) mg/mL	EC50 Ascorbic Acid (PFRAP) mg/mL
35.03 (%)	9.53 ± 0.48 GAE/g dm	1.31 ± 0.06 mg QE/g dm	0.91 ± 0.04 mg/mL	0.03 ± 0.11 mg/mL

**Table 2:** Characterization of the phenolic composition of the *Anacyclus clavatus* flower extract (EtOH:H_2_O, 7:3 *v*/*v*) via HPLC-PDA/ESI-MS.

Peak N°	Compound	t_R_ (min)	UV (nm)	[M-H]^−^	[M + H]^+^	Fragments	Quantity (mg/Kg) Extract ± sd	References
1	Citric acid	1.94	274	191	-	-	Nq	[27]
2	Protocatechuic acid	7.54	204, 259, 293	153	-	-	Nq	[27]
3	Caffeoylquinic acid isomer	9.76	215, 325	353	-	191(-), 179(-)	570.12 ± 9.02	[1,27,28,29,30]
4	Caffeoylquinic acid isomer	11.15	215, 323	353	-	191(-), 179(-)	1373.20 ± 5.54	[1,27,28,29,30]
5	p-Coumaroylquinic acid	12.75	203, 311	337	-	191(-)	500.64 ± 2.13	[1,27]
6	Dicaffeoylquinic acid isomer	13.22	195, 212, 316	515	-	163(+)	479.04 ± 4.52	[1,27,28]
7	Caffeoylquinic acid isomer	13.92	222, 236, 288, 333	353	355	191(-); 181(+)	21,280.34 ± 731.00	[1,27,28,29,30]
8	Caffeoylquinic acid isomer	14.64	217, 325	353	355	191(-); 181(+)	3173.78 ± 68.33	[1,27,28,29,30]
9	Caffeoylquinic acid isomer	15.25	195, 216, 295, 322	353	355	191(-); 181(+)	811.51 ± 30.87	[1,27,28,29,30]
10	Caffeoylquinic acid isomer	15.74	199, 324	353	355	191(-); 181(+)	647.46 ± 39.32	[1,27,28,29,30]
11	p-Coumaroylquinic acid	16.69	211, 223, 311	337	339	191(-); 165(+)	1943.88 ± 62.20	[1,27]
12	Dicaffeoylquinic acid isomer	17.64	215, 321	515	-	163(+); 181(+)	1300.07 ± 54.54	[1,27,28]
13	Feruloylquinic acid	18.16	271, 330	367	369	193(-)	1003.77 ± 34.96	[1]
14	Myricetin-hexoside	19.76	202, 259, 358	479	481	319(+)	2410.94 ± 128.95	[27]
15	Luteolin-dihexoside	20.21	206, 254, 344	623	625	447(+); 287(+)	170.30 ± 11.20	[27]
16	Apigenin 7-O-diglucuronide	22.13	201, 266, 336	621	623	447(+)	420.53 ± 24.21	[27]
17	Apigenin 7-glucuronosyl-glucoside	23.49	202, 266, 336	607	609	269(-); 271(+)	1773.14 ± 81.88	[27]
18	Quercetin O-hexoside	23.80	202, 255, 369	463	465	303(+)	558.33 ± 8.31	[1]
19	Kaempferol-7-O-glucoside	25.55	206, 257, 365	447	449	285 (-)	2508.73 ± 114.46	[27,31]
20	Patuletin 3-O-glucoside	25.91	202, 259, 355	493	495	333(+)	711.03 ± 5.33	[31]
21	Dicaffeoylquinic acid isomer	27.81	216, 325	515	-	181(+)	1449.69 ± 12.16	[32]
22	Dicaffeoylquinic acid isomer	28.36	216, 325	515	-	181(+); 163(+)	6365.15 ± 279.25	[1,27,28]
23	Dicaffeoylquinic acid isomer	28.67	217, 328	515	353	191(-); 163(+) 181(+)	3303.98 ± 146.74	[1,27,28]
24	Apigenin-7-O-glucoside	30.30	201, 266, 336	431	433	271(+)	682.88 ± 25.72	[31]
25	Apigenin 7-O-glucuronide	31.11	201, 266, 336	445	447	269(-)	636.41 ± 23.07	[27]
26	Dicaffeoylquinic acid isomer	32.30	217, 327	515	-	163(+); 181(+)	5815.54 ± 252.78	[1,27,28]
27	Apigenin	43.96	214, 266, 335	269	271	-	80.49 ± 2.05	Std

**Table 3:** MIC (minimum inhibitory concentration) and MBC (minimum bactericidal concentration) values displayed by *Anacyclus clavatus* flower extract against bacterial strains (mg/mL). Values are expressed as means ± SD.

Bacteria	MIC	MBC	MBC/MIC
*Escherichia coli*	20.83 ± 0.12	166.66 ± 0.12	8
*Salmonella typhimirium*	41.66 ± 0.15	166.66 ± 0.17	4
*Staphyloccocus aureus*	20.83 ± 0.20	83.33 ± 0.12	4
*Listeria monocytogenes*	41.66 ± 0.13	166.66 ± 0.16	2

## Data Availability

Not applicable.
